# ^64^Cu-DOTA-trastuzumab PET imaging and HER2 specificity of brain metastases in HER2-positive breast cancer patients

**DOI:** 10.1186/s13550-015-0082-6

**Published:** 2015-03-12

**Authors:** Hiroaki Kurihara, Akinobu Hamada, Masayuki Yoshida, Schuichi Shimma, Jun Hashimoto, Kan Yonemori, Hitomi Tani, Yasuji Miyakita, Yousuke Kanayama, Yasuhiro Wada, Makoto Kodaira, Mayu Yunokawa, Harukaze Yamamoto, Chikako Shimizu, Kazuhiro Takahashi, Yasuyoshi Watanabe, Yasuhiro Fujiwara, Kenji Tamura

**Affiliations:** Department of Diagnostic Radiology, National Cancer Center Hospital, 5-1-1 Tsukiji, Chuo-ku, Tokyo, 104-0045 Japan; Department of Clinical Pharmacology Group for Translational Research Support Core, National Cancer Center Research Institute, Tokyo, Japan; Department of Pathology and Clinical Laboratories, National Cancer Center Hospital, Tokyo, Japan; Department of Breast and Medical Oncology, National Cancer Center Hospital, Tokyo, Japan; Department of Neurosurgery, National Cancer Center Hospital, Tokyo, Japan; RIKEN Center for Life Science Technologies, Hyogo, Japan

**Keywords:** HER2-positive breast cancer, Trastuzumab, PET, ^64^Cu, Brain metastasis

## Abstract

**Background:**

The purpose of this study was to determine whether brain metastases from HER2-positive breast cancer could be detected noninvasively using positron emission tomography (PET) with ^64^Cu-1,4,7,10-tetraazacyclododecane-1,4,7,10-tetraacetic acid (DOTA)-trastuzumab.

**Methods:**

PET was performed on five patients with brain metastases from HER2-positive breast cancer, at 24 or 48 h after the injection of approximately 130 MBq of the probe ^64^Cu-DOTA-trastuzumab. Radioactivity in metastatic brain tumors was evaluated based on PET images in five patients. Autoradiography, immunohistochemistry (IHC), and liquid chromatography-tandem mass spectrometry (LC-MS/MS) analysis were performed in one surgical case to confirm HER2 specificity of ^64^Cu-DOTA-trastuzumab.

**Results:**

Metastatic brain lesions could be visualized by ^64^Cu-DOTA-trastuzumab PET in all of five cases, which might indicated that trastuzumab passes through the blood-brain barrier (BBB). The HER2 specificity of ^64^Cu-DOTA-trastuzumab was demonstrated in one patient by autoradiography, immunohistochemistry, and LC-MS/MS.

**Conclusions:**

Cu-DOTA-trastuzumab PET could be a potential noninvasive procedure for serial identification of metastatic brain lesions in patients with HER2-positive breast cancer.

**Trial registration:**

UMIN000004170

## Background

Many novel molecular targets for anticancer treatment have been discovered. Targeting of HER2 with the monoclonal antibody trastuzumab is a well-established therapeutic strategy for HER2-positive breast cancer in neoadjuvant [1], adjuvant [2], and metastatic settings [3]. Thus, it is important to examine HER2 expression in primary tumor and metastatic sites to determine whether anti-HER2 therapy is indicated.

Although HER2 expression is routinely determined using immunohistochemistry (IHC) or fluorescence *in situ* hybridization (FISH) [4], technical problems can arise when lesions are not easily accessible by core needle biopsy [5]. In addition, HER2 expression can vary during the course of the disease [6] and even among tumor lesions in the same patient [7]. To overcome these problems, a novel molecular imaging technique using positron emission tomography (PET) with ^124^I-, ^89^Zr-, or ^64^Cu-labeled antibodies has been studied for the noninvasive evaluation of HER2 expression [8-10].

In a previous study, we reported the production of ^64^Cu-labeled trastuzumab, specifically ^64^Cu-1,4,7,10-tetraazacyclododecane-1,4,7,10-tetraacetic acid (DOTA)-trastuzumab and that ^64^Cu-DOTA-trastuzumab PET imaging could detect primary HER2-positive breast cancer and metastatic lesions [10]. This imaging technique can be used to serially monitor HER2 tumor status during HER2-targeting treatment and also to evaluate patients with metastatic brain tumors that are not easily accessible by core needle biopsy.

In this study, we demonstrated that ^64^Cu-DOTA-trastuzumab PET imaging could visualize metastatic brain lesions and confirmed the HER2 specificity of ^64^Cu-DOTA-trastuzumab by means of autoradiography, IHC, and liquid chromatography-tandem mass spectrometry (LC-MS/MS).

## Methods

### Patients

The patients included in this study had histologically confirmed invasive HER2-positive (IHC 3+ or FISH-positive) breast carcinoma with at least one site of measurable brain metastasis, Eastern Cooperative Oncology Group performance status (PS) of 0 to 1, adequate organ function (neutrophil count ≥1,500/μL, platelet count ≥75,000/μL, hemoglobin concentration ≥9.0 g/dL, serum bilirubin ≤1.5 mg/dL, AST and ALT ≤100 IU/L, serum creatinine ≤1.5 mg/dL, baseline left ventricular ejection fraction (LVEF) >60%) and were aged between 20 and 75 years. The main exclusion criteria were congestive heart failure, uncontrolled angina pectoris, arrhythmia, symptomatic infectious disease, severe bleeding, pulmonary fibrosis, obstructive bowel disease or severe diarrhea, and symptomatic peripheral or cardiac effusion.

### Preparation of ^64^Cu-DOTA-trastuzumab and PET/CT protocol

^64^Cu-DOTA-trastuzumab was prepared as described previously [10]. Briefly, the ^64^Ni (p, n) ^64^Cu nuclear reaction was performed with 12-MeV proton irradiation using a small medical cyclotron (HM-12S, Sumitomo Heavy Industries Ltd., Tokyo, Japan). The beam current used was approximately 20 μA (3 h). After purification of trastuzumab IgG (Herceptin®; Chugai Pharmaceutical Co., Ltd, Tokyo, Japan) by ultrafiltration (Amicon Ultra 0.5 mL 50 k) with phosphate-buffered saline (PBS), the trastuzumab in PBS was added to DOTA mono *N*-hydroxysuccinimide ester (Macrocyclics Inc., Dallas, TX, USA) and dissolved in water. After incubation at 40°C for 3 h, crude DOTA-trastuzumab was purified with PBS by using a PD-10 column. The PBS buffer, including DOTA-trastuzumab (100 μg, 0.7 nmol), was exchanged for a sodium acetate buffer (100 mM, pH 6.5) by filtration. ^64^Cu-DOTA-trastuzumab was prepared by adding ^64^CuCl_2_ to the acetate buffer solution containing DOTA-trastuzumab and incubating the solution for 1 h at 40°C. The reaction mixture was sterilized by filtration through a 0.22-μm Millex GV filter (Merck Millipore, Billerica, MA, USA). The radiolabeling results revealed that the specific activity and radiochemical purity were approximately 350 GBq/μmol and 98%, respectively. Approximately 500 MBq of the final product was obtained by a single radiosynthesis.

PET/CT studies were performed at 1, 24, and 48 h after ^64^Cu-DOTA-trastuzumab injection with a Discovery 600 (GE Healthcare, Pewaukee, WI, USA), as described previously [10]. PET image evaluation and quantification of the maximum single-voxel standardized uptake value (SUVmax) were performed using AW Volume Share 4.5 software (GE Healthcare). Regions of interest (ROIs) were delineated within the tumor on the PET/CT images, and the SUVmax was determined. For the background uptake, ROIs were placed within the opposite side of normal brain, and the SUVmax was measured.

### Autoradiography, IHC, and LC-MS/MS

One patient (patient no. 5), who had a solitary brain metastasis in the left cerebellum and had planned to undergo surgery, agreed to participate in this study. ^64^Cu-DOTA-trastuzumab injection was performed 1 day prior to PET imaging and subsequent surgical resection. A 20-μm-thick frozen section was immediately prepared from the tumor specimen and used as a sample for autoradiography and IHC, and a 10-μm-thick frozen section was prepared for LC-MS/MS analysis. The residual tissue was fixed and used for routine histopathological diagnosis.

For autoradiography, the frozen section was placed on an imaging plate (BAS IP SR 2025, GE healthcare) for 48 h at 4°C to detect the location of radioactivity in the specimen, and the exposed imaging plate then was read with a FLA 9000 laser scanner system (GE Healthcare). IHC staining was also performed on the cryosection adjacent to that used for autoradiography to assess the spatial distribution of ^64^Cu-DOTA-trastuzumab. The HercepTest™ kit (DAKO, Glostrup, Denmark) was used for detection of HER2-positive tumor cells. Digitization and registration of corresponding images from the autoradiogram and IHC were performed using a microcomputer system and ImageQuant TL Analysis Toolbox software (GE Healthcare).

**Figure 1:**
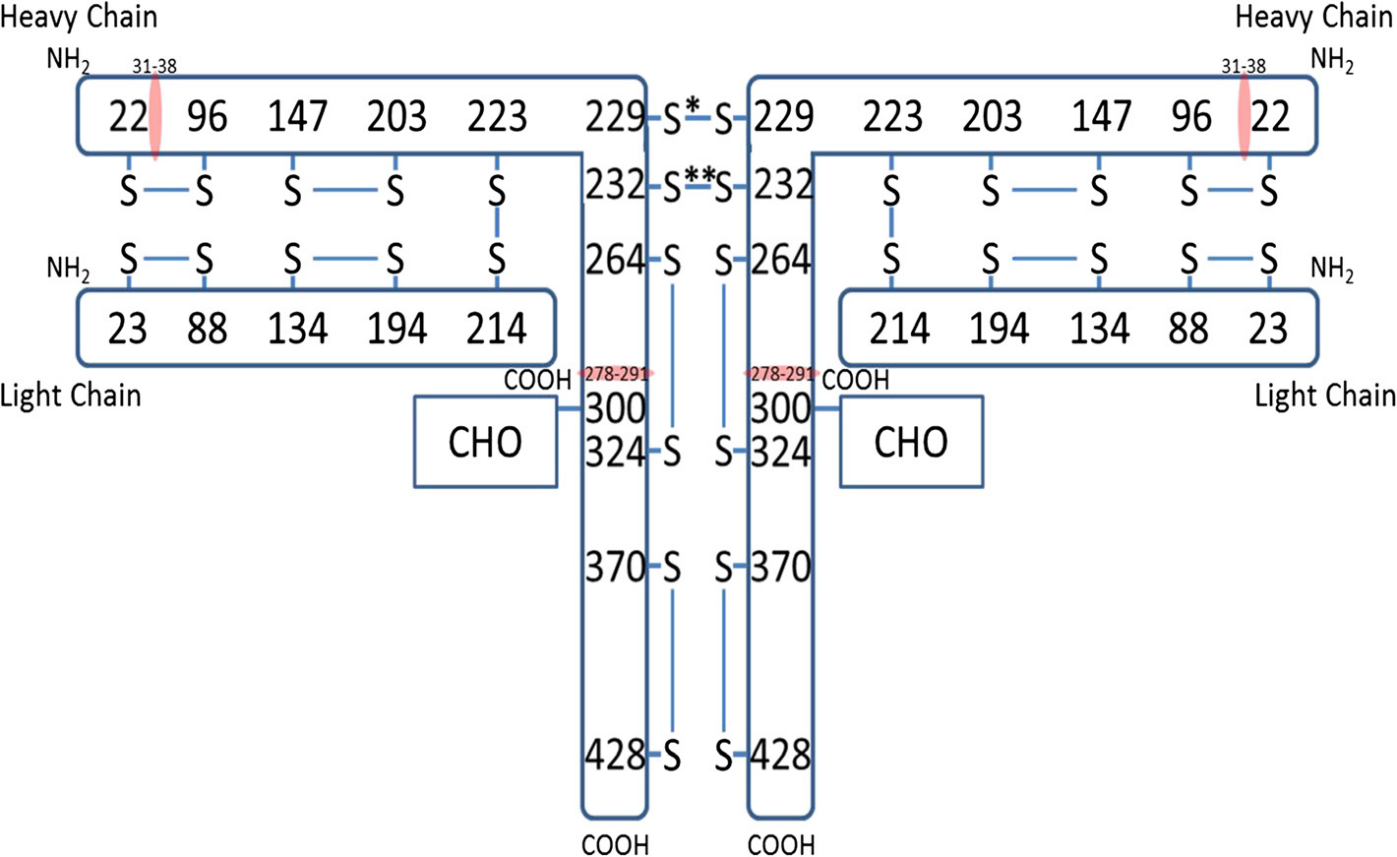
**Schematic structure of trastuzumab antibody and the locations of FCR and CDR (red).**

To measure the exposure levels of trastuzumab in tumor and non-tumor tissue sites, the relative amount of complementarity-determining region (CDR) analyte was estimated by using a laser capture micro-dissection system (LCM; LMD7000, Leica, Buffalo Grove, IL, USA) and LC-MS/MS system (LC; Nexera HPLC system, Shimadzu, Chestnut Ridge, NY, USA; MS; QTRAP 4500, AB SCIEX, Framingham, MA, USA). The schematic structure of the trastuzumab antibody and the structural locations of the CDR and Fc receptor (FCR) are shown in Figure 1. In brief, the dehydrated tumor sections were stained with hematoxylin and eosin (HE). Individual tumor and non-tumor regions were visually dissected using LCM. The captured tissues were dissolved in 8 M urea, and the amount of protein was analyzed by BCA protein assay kit (Thermo Fisher Scientific Inc., Waltham, MA, USA). The crude solutions were treated with reduction by tris-(2-corboxyethyl)-phosphine hydrochloride, alkylation by iodoacetamide, and fragmentation by trypsin at 37°C for 12 h. The peptide-containing reaction mixtures were dissolved in 0.1% formic acid and used as samples for LC-MS/MS. Since the CDR in the trastuzumab heavy chain was digested as a specific peptide (DTYIHWVR, *m/z* 545.3 detected as a doubly charged ion), this peptide was set as the target analyte in LC-MS/MS (MRM mode, transition was 545.3 > 597.3).

### General

All chemical reagents were obtained from commercial sources. This study was conducted according to a protocol approved by the institutional review board and independent ethics committee of the National Cancer Center Hospital. All patients signed a written informed consent form.

## Results and discussion

### Patient characteristics

Between December 2010 and November 2013, five patients were enrolled in the current study. The median age of the patients was 59 years. Histologically, tumors were invasive ductal breast carcinoma, of either the solid tubular or scirrhous type. Four patients had HER2-positive tumors that were IHC 3+, whereas one patient had a HER2-positive tumor that was both IHC 2+ and FISH positive. One patient (patient no. 5) with a metastatic brain tumor underwent a 24-h PET imaging study, after which the tumor was surgically resected (Table 1).

**Table 1:** **Patient characteristics**

**Number**	**Age (y)**	**Histology**	**IHC**	**History of trastuzumab treatment**	**Number of lesions (** ***φ*** **<1 cm) detected by MRI/CT/** ^**64**^ **Cu**	**Number of lesions (** ***φ*** **>1 cm) detected by MRI/CT/** ^**64**^ **Cu**
1	73	IDC-st	3+	Weekly	2/0/0	4/4/4
2	75	IDC-sc	3+	Weekly	0/0/0	1/0/1
3	65	IDC-sc	3+	Weekly	8/0/2	0/0/0
4	54	IDC-sc	3+	Tri-weekly	3/0/1	1/1/1
5	61	IDC-sc	3+	Weekly	0/0/0	1/1/1

y, years; IDC-st, invasive ductal carcinoma-solid tubular; IDC-sc, invasive ductal carcinoma-scirrhous; ^64^Cu, ^64^Cu-DOTA-trastuzumab PET; Weekly, 2 mg/kg/week; Tri-weekly, 8 mg/kg/3 week.

The mean and standard deviation of the administrated mass of ^64^Cu-DOTA-trastuzumab was 74.4 ± 6.8 μg (range, 67.8 to 80.6 μg). The mean administered activity was 141 ± 8 MBq (range, 133 to 148 MBq).

The administration of ^64^Cu-DOTA-trastuzumab was well tolerated by all subjects. No infusion- or drug-related adverse events were reported in any of the patients. No clinically important trends indicative of safety issues were noted in the laboratory parameters, vital signs, or electrocardiogram parameters.

### PET imaging

**Figure 2:**
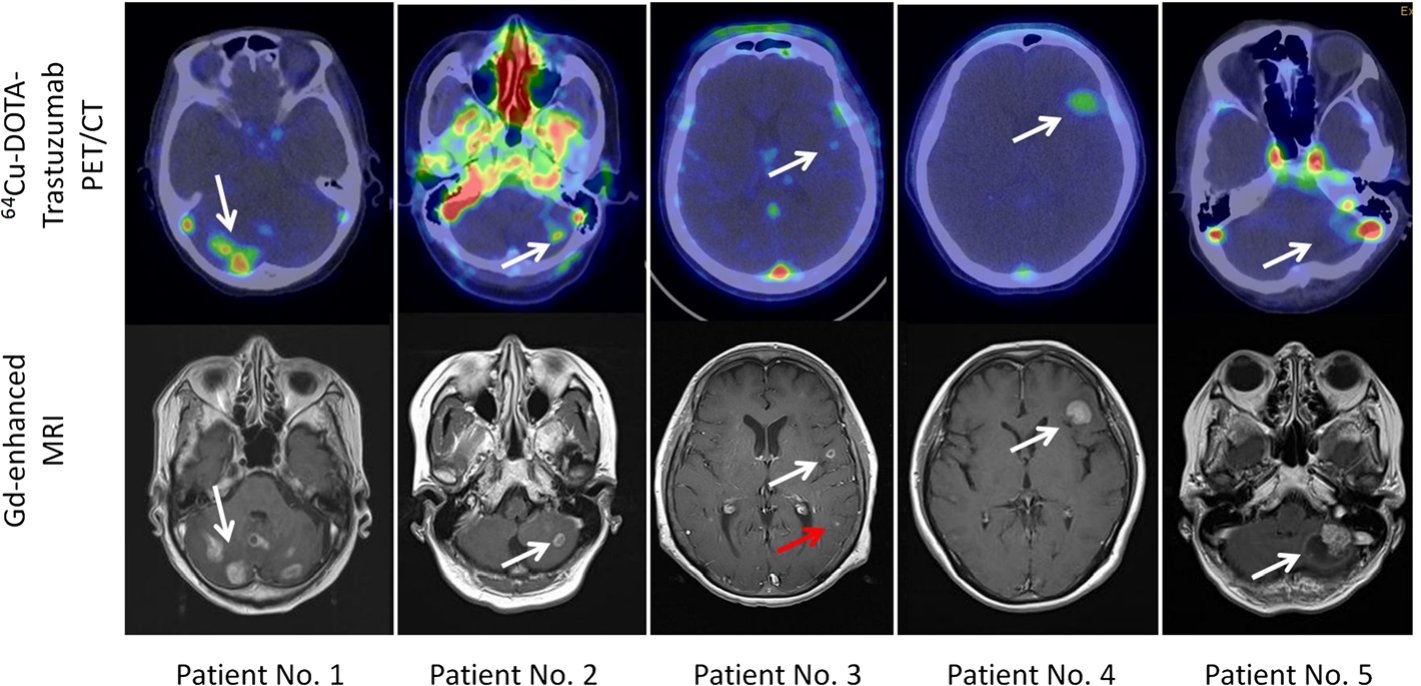
^**64**^ **Cu-DOTA-trastuzumab PET images of metastatic brain tumors in patients with HER2-positive primary breast tumors.** The white arrows show the metastatic brain tumors. Upper panels: ^64^Cu-DOTA-trastuzumab PET images; lower panels: Gd-DTPA-enhanced T1-weighted MRI images. White arrows indicate metastatic brain lesions detectable by both MRI and ^64^Cu-DOTA-trastuzumab PET, and a red arrow indicates a lesion detectable by MRI but not by ^64^Cu-DOTA-trastuzumab PET. In patient no. 2 PET image, non-specific high uptake in blood was noted.

In 5 patients, 20 metastatic brain lesions including 7 lesions larger than 1 cm diameter and 13 lesions smaller than 1 cm diameter were previously identified by CT or magnetic resonance imaging (MRI). In this study, all of the HER2-positive brain lesions larger than 1 cm diameter could be seen on the ^64^Cu-DOTA-trastuzumab PET scan at 24 and 48 h after injection. On the other hand, 10 of 13 metastatic brain tumors smaller than 1 cm diameter were not easily identified by ^64^Cu-DOTA-trastuzumab PET imaging (Table 1). Typical images of the lesions that could be detected by both MRI and ^64^Cu-DOTA-trastuzumab PET, and the lesion that could not be identified by ^64^Cu-DOTA-trastuzumab PET were demonstrated in Figure 2 (white arrow and red arrow). The limited spatial resolution of PET leads to partial volume effects and, consequently, to limited signal recovery for SUVmax, which is affected for small structures. This could be one of the explanations why tumors smaller than 1 cm diameter were not identified by ^64^Cu-DOTA-trastuzumab PET. The location, SUVmax, background uptake, and TNR of each lesion visualized by ^64^Cu-DOTA-trastuzumab PET are summarized in Table 2. Brain tumor accumulation of ^64^Cu-DOTA-trastuzumab was higher at 48 h than at 24 h after injection in four cases. In the other one case, ^64^Cu-DOTA-trastuzumab PET imaging at 48 h after injection was not carried out because of surgical resection. Since non-specific background uptakes in corresponding normal brain tissues were very low, tumor-to-normal tissue count ratios (TNR) were high (Table 2), resulting in good contrast for detecting brain metastases. The average TNR values at 24 h and 48 h were 6.7 ± 2.1 and 9.8 ± 3.3, respectively.

**Table 2:** **Accumulation of** ^**64**^ **Cu-DOTA-trastuzumab in brain tumor**

**Patient number**	**Lesion number**	**Location**	**SUVmax (tumor/BG)**		**TNR**	
			**24 h**	**48 h**	**24 h**	**48 h**
1	1	Rt-cerebellum	1.4/0.2	2.1/0.2	9.3	12.5
	2	Rt-cerebellum	1.3/0.2	2.1/0.2	8.7	12.4
	3	Vermis	1.4/0.2	2.1/0.2	9.1	12.4
	4	Lt-cerebellum	1.1/0.2	1.6/0.2	7.1	11.8
2	5	Lt-cerebellum	1.2/0.1	2.1/0.2	8.2	12.2
3	6	Lt-frontal	0.7/0.2	1.0/0.2	4.1	5.5
	7	Lt-frontal	0.6/0.2	0.7/0.2	3.3	4.1
4	8	Lt-frontal	0.9/0.2	1.6/0.2	5.8	9.2
	9	Rt-cerebellum	0.8/0.2	1.2/0.2	5.1	7.8
5	10	Lt-cerebellum	1.1/0.2	NA/NA	6.1	NA

^64^Cu-DOTA-trastuzumab PET imaging at 48 h after injection was not carried out because of surgical resection.

BG, background; NA, inpatient no. 5.

The balance between the uptake in the tumor, blood clearance of injected ^64^Cu-DOTA-trastuzumab, and the radioactive decay of ^64^Cu indicate the plausible imaging time of 48 h after the injection. Compared to an optimal imaging time of 4 to 5 days after ^89^Zr-trastuzumab injection [9], imaging time of 48 h after ^64^Cu-DOTA-trastuzumab injection seems to be more acceptable in clinical practice. Because of its relatively longer half-life, ^89^Zn-trastuzumab provides clearer images as the patient can be imaged at longer time points; however, it induces higher radiation exposure. On the other hand, the shorter half-life of ^64^Cu induces lower radiation exposure; however, it provides images with non-specific activity in the blood [10]. One possible approach to decrease the high uptake of the probe by the liver could be to use cold trastuzumab with ^64^Cu-DOTA-trastuzumab injection. However, adequate interval from pre-dosing of the cold trastuzumab should be determined in the future study.

### HER2 specificity of ^64^Cu-DOTA-trastuzumab in a human subject

**Figure 3:**
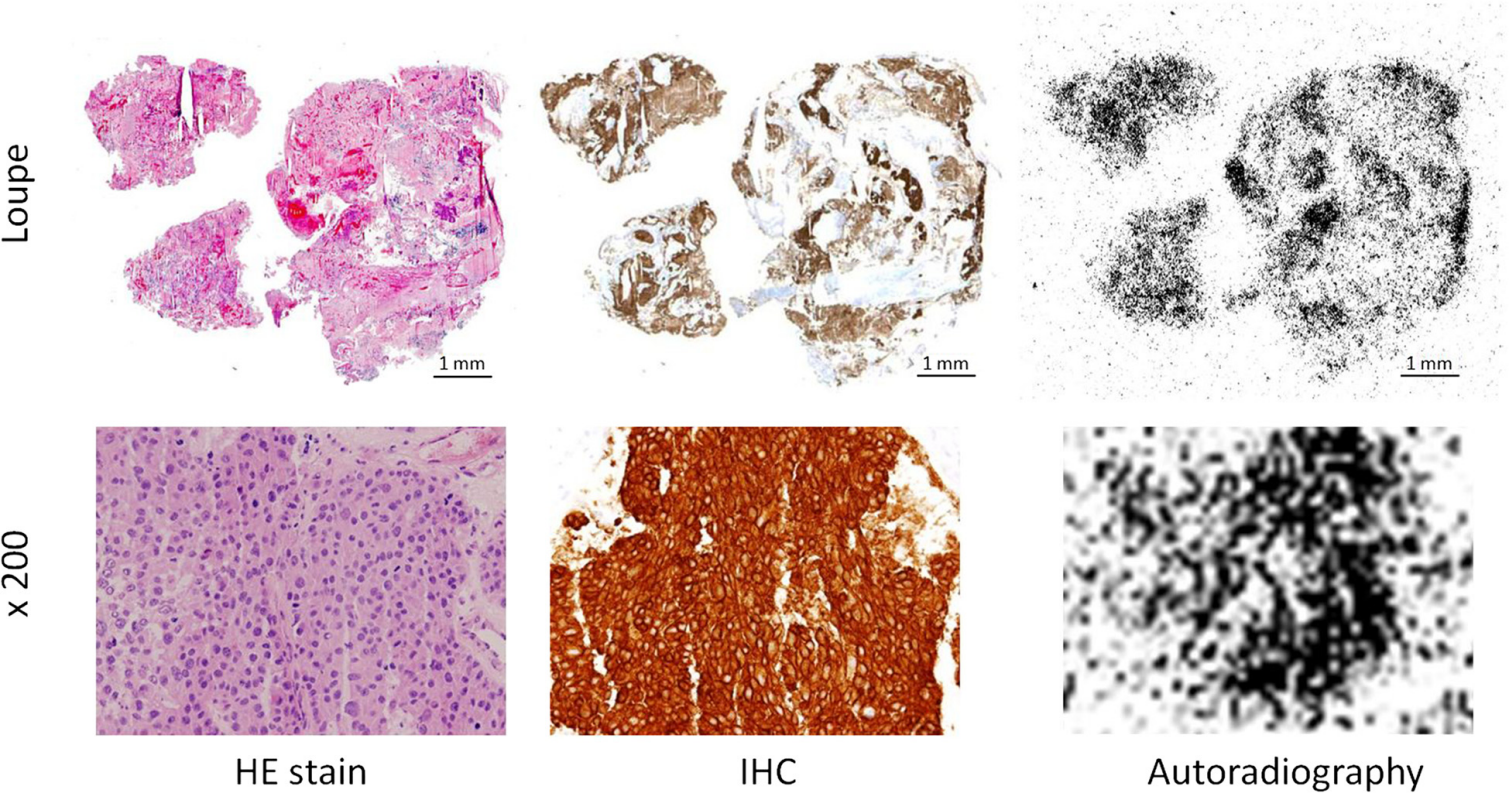
**Histological distributions of** ^**64**^ **Cu-radioactivity and HER2-positive tumor cells.** Left column: HE staining; middle column: IHC; right column: autoradiography. Loupe images (upper panels) show identical distribution of radioactivity and location of HER2-positive tumor cells for HE stain, IHC, and autoradiography samples. Magnified images (lower panels, ×200) confirmed the radioactivity and HER2-positive status of tumor cells.

In patient no. 5, a metastatic brain tumor of 2.8 × 3.1 cm was visualized by ^64^Cu-DOTA-trastuzumab PET imaging at 24 h after injection (Figure 2). PET imaging was not performed at 48 h after injection in this case because of the scheduled surgical operation. An autoradiogram of the frozen section prepared from the removed brain tumor specimen revealed high accumulation in the area where HER2-positive cells were seen by IHC (Figure 3), confirming the HER2 specificity of ^64^Cu-DOTA-trastuzumab PET imaging in a human subject.

**Figure 4:**
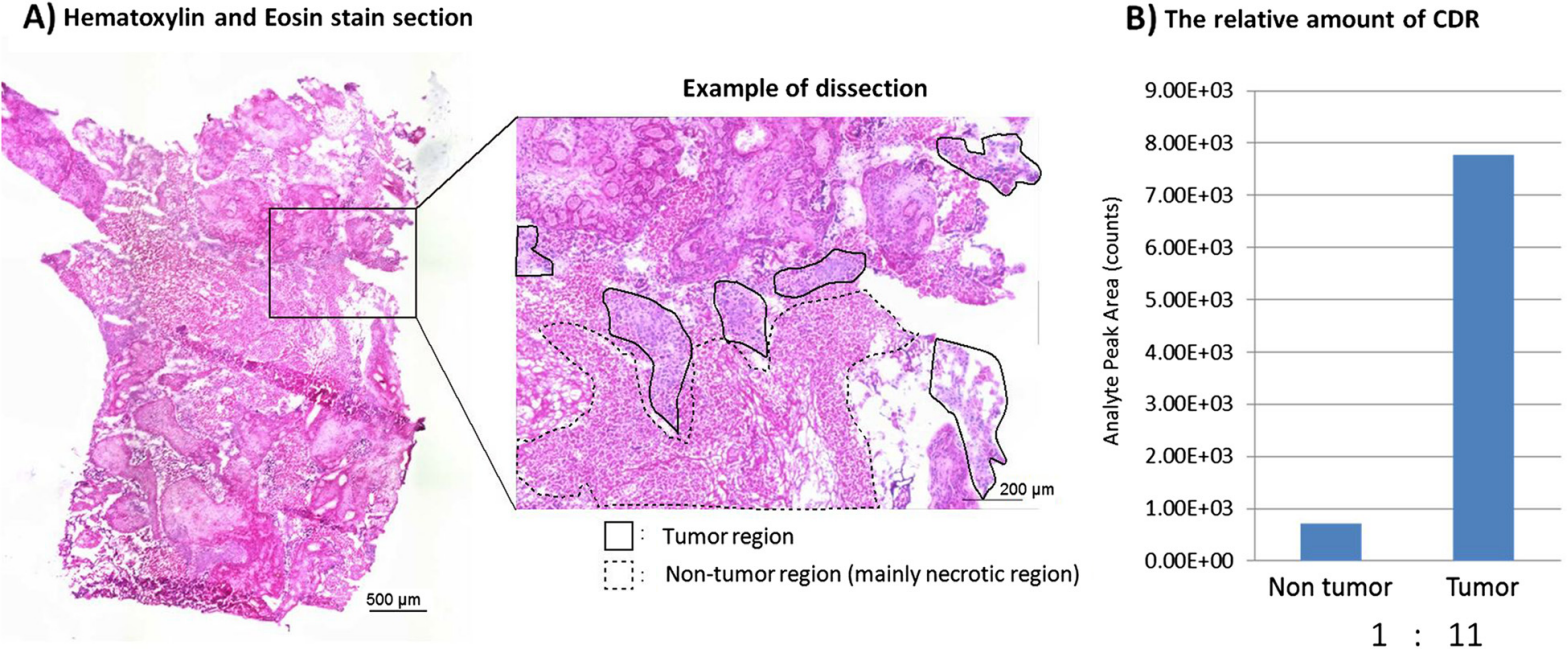
**Relative amount of trastuzumab-specific CDR in tumor cell regions and non-tumor cell regions. (A)** HE staining. Tumor regions (inside solid lines) and non-tumor regions (inside dashed lines) were dissected individually by LCM and collected. **(B)** The relative amount of trastuzumab-specific CDR. LC-MS/MS analysis revealed that the target peptide (DTYIHWVR, *m/z* 545.3 detected as a doubly charged ion) in tumor cell regions was 11-fold higher than that in non-tumor cell regions.

For LC-MS/MS analysis, the tumor and non-tumor areas were dissected by LCM (Figure 4A). The non-tumor area consisted mainly of necrotic tissue. The protein contents in the tumor and non-tumor areas were 26 μg and 21 μg by BCA assay, respectively. The analyte peak area of CDR in the tumor area was significantly higher than that in the non-tumor area. The CDR analyte count ratio for tumor versus non-tumor areas was calculated to be 11:1 (Figure 4B).

This study accumulated safety data and demonstrated clinical cases of brain metastases visualized by ^64^Cu-DOTA-trastuzumab PET imaging. We confirmed the HER2 specificity of ^64^Cu-DOTA-trastuzumab by autoradiography, IHC, and LC-MS/MS in one case of metastatic brain tumor resection.

In clinical settings, it is not possible to obtain metastatic brain tissue samples without surgery. However, brain metastasis is documented in 10% to 16% of patients with metastatic breast cancer during the course of their disease [11,12] and in 40% of patients during the course of herceptin treatment [13]. The IHC profiles of metastatic brain tumors from breast cancer have been reported to differ from the primary site in a certain frequency [14]. Some cases of HER2-positive breast cancer may have HER2-negative brain metastasis, and the converse is also true. Furthermore, since brain metastases generally occur late in the course of metastatic breast cancer, one result of the improving overall survival rate in these patients is that the incidence of brain metastasis will likely increase [15]. Knowing the HER2 status of a metastatic brain tumor would assist in identifying the best additional systemic treatment. ^64^Cu-DOTA-trastuzumab PET imaging is a potential solution for noninvasively and serially evaluating brain tumor HER2 status.

Although it is generally considered that trastuzumab poorly penetrates the blood-brain barrier (BBB), we were able to visualize brain lesions in this study. Therapeutic antibodies such as trastuzumab are thought to be too large or hydrophilic to cross the BBB [16,17]. In addition, penetration of the tumor by monoclonal antibodies may be hindered by increased intratumoral interstitial pressure [18-20]. In this study, however, high CNS penetration of ^64^Cu-DOTA-trastuzumab was demonstrated by LC-MS/MS with an 11-fold higher CDR analyte count in the tumor cell region than the non-tumor cell region and by PET imaging with a TNR of 5 to 12 at 48 h after injection. Another study using ^89^Zr-trastuzumab PET imaging also demonstrated CNS penetration in patients with metastatic brain tumors, with an 18-fold higher uptake in brain tumors than in normal brain tissue at 4 to 7 days after injection [9]. Some researchers have reported that this increased penetration is likely due to disruption or compromise of the BBB at the site of brain metastasis by the metastatic tumor itself or by cancer therapies such as radiotherapy [21,22], though one report suggested that the BBB in HER2-positive tumors was disrupted less than in triple-negative or basal-type breast cancer [23]. Another potential mechanism for transporting trastuzumab across the BBB is the FCR for immunoglobulin G, which expressed on vessels in the brain [24]. However, further studies are needed to clarify the mechanisms involved in transportation across the BBB in tumor sites.

PET imaging with ^64^Cu-DOTA-trastuzumab has the potential to characterize HER2 status using a potent quantitative biomarker that can predict the biological effect of anti-HER2 antibodies. This information might help clinicians determine the optimal HER2 inhibitor therapy for each individual. In this study there was high accumulation in the normal liver. The SUVmax in the liver at 24 h and 48 h post-injection ranged 5.1 to 8.2 and 5.2 to 8.0, respectively, which may interrupt to detect occult liver metastasis. The high accumulation in normal liver is likely due to high expression of FCR on liver sinusoidal endothelial cell [25]. Another research group reported that 50 mg of trastuzumab reduced liver uptake of ^64^Cu-DOTA-trastuzumab in HER2-positive metastatic breast cancer [26]. These findings demonstrate that ^64^Cu-DOTA-trastuzumab PET/CT might be applicable not only for breast cancer, but for other kinds of HER2-positive malignancies as well.

In this study, HER2 specificity of ^64^Cu-DOTA-trastuzumab was demonstrated for one case. The HER2-positive status of the tumor was confirmed by IHC, high radioactivity was shown in the area of HER2-positive tumor cells by autoradiography, LC-MS/MS revealed an 11-fold higher trastuzumab CDR amount in the tumor cell region than in the non-tumor region, and the average TNR of ^64^Cu-DOTA-trastuzumab PET imaging at 48 h was 9.8 ± 3.3. These results strongly suggest the HER2 specificity of ^64^Cu-DOTA-trastuzumab imaging. However, for the other four cases, we could not confirm the HER2 status of metastatic brain tumors because they did not undergo surgery. This is a potential weakness of our study, but in general, it is difficult to sample metastatic brain tumors by surgical operation because patients with HER2-positive breast cancer and metastatic brain tumors are usually treated with systemic chemotherapy. To confirm HER2 specificity of ^64^Cu-DOTA-trastuzumab PET imaging in humans, more cases of both HER2-positive and HER2-negative tumors are required, along with further study comparing the results of PET imaging with HER2 status.

## Conclusions

^64^Cu-DOTA-trastuzumab PET imaging was safe and feasible for outpatients. This technique can be used to visualize metastatic brain lesions in HER2-positive breast cancer patients. HER2 specificity of ^64^Cu-DOTA-trastuzumab was demonstrated in a surgical resection case by means of autoradiography, IHC, and LC-MS/MS. To evaluate, ^64^Cu-DOTA-trastuzumab is a promising candidate for the noninvasive and serial evaluation of HER2 status in metastatic brain tumors.

## Abbreviations

BBB: blood-brain barrier
CDR: complementarity-determining region
DOTA: 1,4,7,10-tetraazacyclododecane-1,4,7,10-tetraacetic acid
FCR: Fc receptor
FISH: fluorescence *in situ* hybridization
HE: hematoxylin and eosin
IHC: immunohistochemistry
LCM: laser capture micro-dissection system
LC-MS/MS: liquid chromatography-tandem mass spectrometry
LVEF: left ventricular ejection fraction
MRI: magnetic resonance imaging
PET: positron emission tomography
PS: Eastern Cooperative Oncology Group performance status
ROIs: regions of interest
SUVmax: maximum single-voxel standardized uptake value
TNR: tumor-to-normal tissue count ratios
